# Nodoventricular pathway-mediated supraventricular tachycardia mimicking typical atrioventricular nodal reentrant tachycardia: Diagnostic and therapeutic challenges

**DOI:** 10.1016/j.hrcr.2025.10.017

**Published:** 2025-10-21

**Authors:** Mohammad Iqbal, Raymond Pranata, Setiawan Widodo, Arsha Pramudya, Giky Karwiky, Chaerul Achmad

**Affiliations:** 1 Department of Cardiology and Vascular Medicine, Faculty of Medicine, Universitas Padjadjaran, Hasan Sadikin General Hospital, Bandung, Indonesia; 2 Hasna Medika Cardiovascular Hospital, Cirebon, Indonesia

**Keywords:** Nodoventricular pathway, Supraventricular tachycardia, AVNRT, Ablation, Accessory pathway

**Key Teaching Points**

• Despite the presence of classic atrioventricular nodal reentrant tachycardia features, multiple failed ablation attempts should prompt consideration of other possible causes.
• Pacing at both the basal and apical areas can help confirm the presence of an accessory pathway. This is demonstrated by “A” advancement despite ventricular not fully captured and a shorter post-pacing interval minus tachycardia cycle length interval at the basal area compared with the apical area.
• QRS fusion during entrainment can help differentiate a nodoventricular pathway from a nodofascicular pathway.

## Introduction

Atrioventricular nodal reentrant tachycardia (AVNRT) is a common cause of supraventricular tachycardia (SVT), with typical AVNRT involving a slow-fast pathway characterized by an AH jump and ventriculoatrial (VA) conduction of <70 ms.^1^ In contrast, SVT mediated by the nodoventricular or nodofascicular pathway with VA <70 ms initiated by AH jump is rare, and its electrophysiological (EP) characteristics remain poorly understood.^1^^,^^2^ Diagnostic maneuvers are essential for confirming the diagnosis and excluding typical AVNRT, and a cautious, stepwise ablation approach is necessary to minimize complications.

We report a case of SVT initially suggestive of typical AVNRT, which was ultimately found to be mediated by a nodoventricular pathway. This case highlights the EP differences between typical AVNRT, and nodoventricular and nodofascicular-mediated SVT, underscoring their importance in accurate diagnosis.

## Case report

A 42-year-old woman presented with recurrent palpitations. The baseline electrocardiogram (ECG) was normal. One quadripolar catheter was introduced on His, one duo-decapolar catheter was introduced along the low right atrial crista (DD19-20) to distal coronary sinus (DD1-2), and an ablation catheter was placed on RV for the first time for EP study. An EP study revealed signs of slow-fast AVNRT with an AH jump (S1S2 600/280 ms) that initiated the tachycardia, VA <70 ms and post-pacing interval minus tachycardia cycle length (PPI-TCL) >115 ms from apical RV. Slow-pathway ablation was performed, and the electrogram (EGM) demonstrated slow junctional rhythm during ablation. However, the tachycardia remained inducible despite multiple ablation attempts.

**Figure 1:**
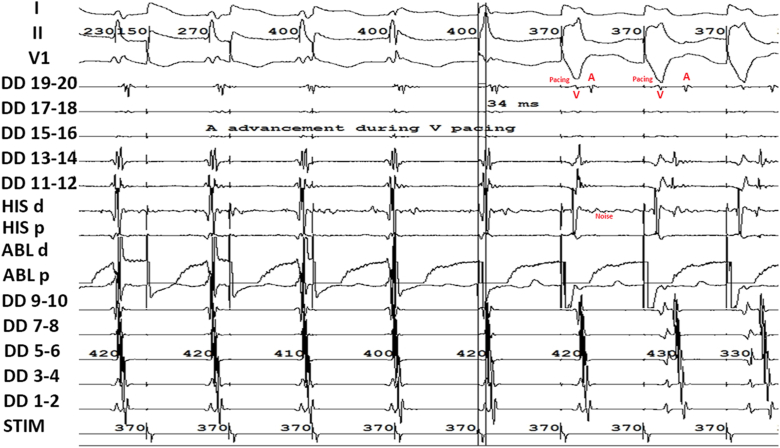
EGM showing A advancement during V pacing before complete entrainment V capture. EGM **=** electrogram.

**Figure 2:**
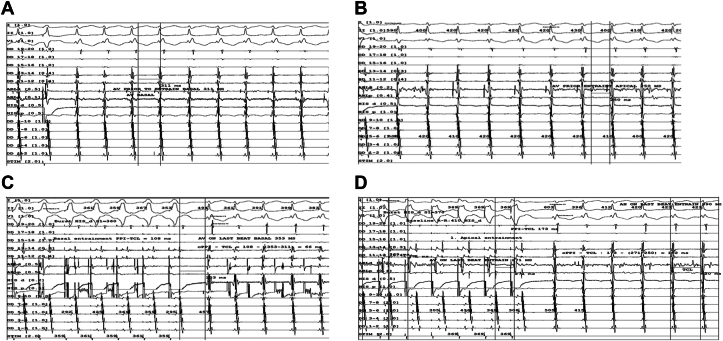
EGM showing AH prior to entrainment at basal **(A)** and apical **(B)**. Entrainment showing a shorter corrected post-pacing interval minus tachycardia cycle length (cPPI-TCL) at the basal **(C)** compared with apical **(D)** site. EGM **=** electrogram.

**Figure 3:**
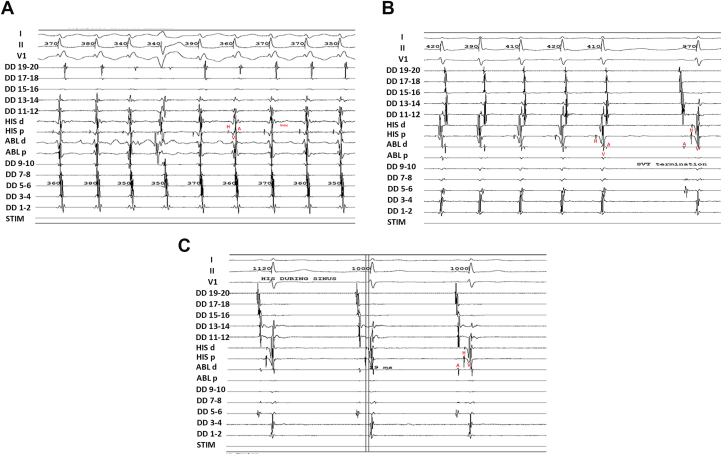
EGM showing supraventricular tachycardia (VA <70 ms) **(A)** and termination of the tachycardia was ended with A **(B)**. AH interval of <40 ms during tachycardia and an HV interval during sinus rhythm equal to that during tachycardia. **(C)** showed antegrade activation during sinus rhythm. EGM **=** electrogram; VA = ventricular tachycardia.

We then performed ventricular overdrive pacing from basal and apical RV. “A” advancement was observed before complete entrainment capture, and ventricular fusion was noted before full ventricular capture (Figure 1). EGM showing AH prior to entrainment at basal and apical were reported in (Figure 2A–B). Entrainment was performed at both sites, showing a shorter corrected post-pacing interval minus tachycardia cycle length (cPPI-TCL) at the basal site (66 ms at the basal site vs 152 ms at the apical site) (Figure 2C–D). EGM showed AH interval of <40 ms during tachycardia and an HV interval during sinus rhythm equal to that during tachycardia (Figure 3A–C). His-refractory premature ventricular contraction (PVC) from RV basal and apical did not reset the tachycardia. These patterns suggested a nodoventricular pathway with bystander dual AV nodal physiology (Table 1).

**Table 1:** Findings during electrophysiology study

Findings	Interpretation
“A” advancement during pacing despite partial ventricular capture	Suggestive of the presence of accessory pathway rather than AVNRT
QRS fusion during entrainment	Suggestive of nodoventricular rather than nodofascicular pathway
Corrected PPI-TCL at basal is shorter compared to apical	Suggestive of the presence of accessory pathway rather than AVNRT
Discrete potential at the ablation site (possibly accessory pathway potential) inside the coronary sinus	Suggestive of the presence of accessory pathway rather than AVNRT

AVNRT = Atrioventricular nodal reentrant tachycardia; PPI-TCL = post-pacing interval minus tachycardia cycle length.

**Figure 4:**
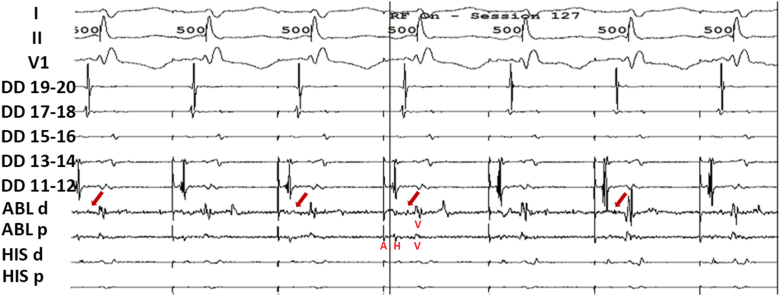
EGM showing discrete potential at the ablation site (possibly accessory pathway potential) (*red arrow*) inside the coronary sinus. EGM **=** electrogram.

**Figure 5:**
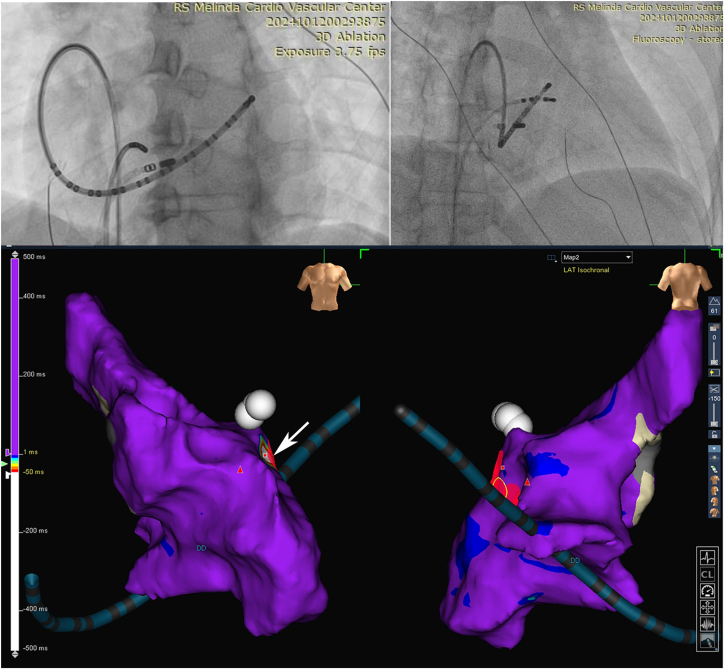
Fluoroscopy and 3D electroanatomic mapping during tachycardia. Fluoroscopy showing the RAO and LAO position of ablation catheter in the coronary sinus and 3D electroanatomic activation mapping during tachycardia showing the ablation target. The *white arrow* points to the *red box* that indicates the site of earliest activation mapping. 3D = 3-dimensional; LAO = left anterior oblique; RAO = right anterior oblique.

Three-dimensional activation mapping during tachycardia identified the earliest atrial activation in the coronary sinus, confirming a nodoventricular pathway. EGM showing discrete potential at the ablation site (possibly accessory pathway potential) (*red arrow*) inside the coronary sinus (Figure 4). Ablation was performed 2 cm from the coronary sinus ostium near the left inferior extension (LIE) of the AV node (Figure 5); the AH jump persisted, but tachycardia was no longer inducible, both during infusion and washout isoproterenol.

## Discussion

This case initially presented as typical AVNRT based on standard EP findings followed by slow-pathway ablation. However, further testing revealed the presence of a nodoventricular pathway following AH jump. Despite showing classical AVNRT findings such as AH jump, VA 0 ms, and PPI-TCL >115 ms from apical RV entrainment; repeated right inferior extension (RIE) pathway ablations failed to terminate tachycardia. Therefore, other possible causes should be considered. We reevaluated our apical RV entrainment, which showed “A” advancement despite partial ventricular capture, indicating the presence of accessory pathway. Certain atypical forms of AVNRT use lower septal slow-pathway inputs that originate from right or left inferior nodal extensions, which can produce atrial activation patterns and ventricular–atrial timing that closely resemble tachycardias mediated by true nodoventricular pathways. In these variants, the earliest retrograde atrial signal may occur near the coronary sinus ostium or low septum, the RP interval may be relatively long, and responses to pacing maneuvers (such as His-refractory PVCs) can sometimes mimic the behavior of a nodoventricular connection.^3^, ^4^, ^5^, ^6^ A diagnosis of AVNRT with unusual behavior cannot be fully excluded and our findings were most compatible with, but not diagnostic of, the presence of nodoventricular pathway.

Entrainment at basal and apical sites revealed a shorter cPPI-TCL interval at the basal region compared to the apical region, indicating its involvement in the tachycardia circuit. This finding effectively excludes AVNRT, which is typically characterized by a PPI-TCL interval >120 ms.^7^ Although literature on nodoventricular and nodofascicular pathways is limited, our findings support previous reports emphasizing the importance of specific electrophysiologic maneuvers to localize accessory pathways.

In a series of 9 patients with nodoventricular (7 patients) and nodofascicular (2 patients) pathways, 88% were found to have dual AV nodal physiology.^6^ The AH jump in this patient acted as critical time for reentrant tachycardia. Still, it was not the main substrate for the tachycardia as evidenced by non-inducibility during and after isoproterenol provocation post-accessory pathway ablation. Prior studies have identified diagnostic criteria distinguishing nodoventricular/nodofascicular pathways from AVNRT, including PPI-TCL <115 ms with 100% positive predictive value. These characteristics also observed in our patient, corroborating the previous study and reaffirming the diagnostic features of nodoventricular/fascicular pathways rather than AVNRT. However, it is noteworthy that during apical pacing in our case, cPPI-TCL was prolonged. While earlier studies have proposed a cutoff value of 125 ms for distinguishing such pathways, another case report had documented values exceeding this threshold.^6^^,^^8^ This discrepancy may suggest the existence of nodoventricular pathways with cPPI-TCL values greater than 125 ms, or it may reflect suboptimal apical pacing that does not accurately capture the true apex. PVC delivered when His Bundle was committed failed to advance or terminate the tachycardia. Although this maneuver has 100% specificity, it does not have 100% sensitivity; therefore, a negative result does not rule out the presence of an accessory pathway.^9^^,^^10^ Nodoventricular/fascicular tachycardia may present as narrow complex tachycardia with VA dissociation; however, we did not observe VA dissociation in this patient.^11^

There was “A” advancement before complete entrainment capture, indicating a presence of accessory pathway. Entrainment showed ventricular fusion (Figure 1), indicating that the pathway involves the ventricle, supporting the diagnosis of a nodoventricular pathway rather than nodofascicular pathway.^6^ A case series has indicated that slow-pathway ablation may be sufficient for nodoventricular or nodofascicular tachycardia in some instances.^1^ Given the proximity of these pathways to the conduction system, ablation of the slow pathway can be considered as an initial approach if it successfully renders the tachycardia non-inducible. However, in our case, despite repeated successful ablations of the RIE, the tachycardia remained inducible. Left-sided nodofascicular and nodoventricular accessory pathways often connect the ventricles to the coronary sinus musculature near the region of the coronary sinus ostium.^12^ We employed three-dimensional mapping to localize the accessory pathway at the coronary sinus ostium near the LIE of the AV node. Ablation in this region successfully eliminated the pathway. Following the procedure, aggressive pacing failed to induce tachycardia, confirming procedural success.

## Conclusion

We presented a case of SVT initially suggestive of typical AVNRT by EP study, which was ultimately determined to be mediated by a nodoventricular pathway. Although the diagnosis of AVNRT with unusual behavior cannot be fully excluded the findings were most compatible with the presence of nodoventricular pathway. When slow-pathway ablation fails to terminate the tachycardia, further maneuver is important to find another mechanism of tachycardia.

## Disclosures

The authors have no conflicts of interest to disclose.
